# Does the Mode of Exercise Influence the Benefits Obtained by Green Exercise?

**DOI:** 10.3390/ijerph16163004

**Published:** 2019-08-20

**Authors:** Matthew Fraser, Sarah-Anne Munoz, Sandra MacRury

**Affiliations:** Department of Rural Health and Wellbeing, University of the Highlands and Islands, Inverness IV3 3JH, UK

**Keywords:** green exercise, walking, golf, cognitions, nature

## Abstract

Green exercise studies have tended to use walking as a modality of exercise to establish benefits to mental health. Whether green exercise benefits translate into different forms of green exercise has been deemed an important research gap. A mixed-methods study design was used to compare psychological responses between two forms of green exercise; golf and walking. A total of 20 participants (10 in each group), with a range of ages and experience were recruited to take part in the study. Participants in the walking condition exhibited significantly greater levels of dissociative cognitions than golf condition participants. Consequently, only the walking condition significantly improved in a directed attention test. Results from the Exercise-Induced Feeling Inventory questionnaire found the walking condition demonstrated increases in all four subscales, whereas the golf condition showed no significant improvements. Based on the findings from the qualitative analysis, distinct differences were seen with regards to the perception of the environment. Participants in the golf condition noted natural elements as obstacles to effective performance, whereas the walking group noted natural stimuli as evoking positive feelings. In agreement with the Attention Restoration Theory, the current study demonstrates that the benefits of green exercise are somewhat reduced when greater levels of directed attention towards the activity are exhibited during green exercise.

## 1. Introduction

The psychological benefits of conducting physical activity in the presence of nature, termed ‘green exercise’ have been well established over the past two decades of research [1,2]. The majority of green exercise studies have examined psychological effects. Previous studies investigating green exercise have found numerous synergistic benefits to conducting exercise in natural areas including; decreased levels of depression [3], short-term reductions in anxiety [4], decreased mood disturbance [5], improved affective responses [6,7], greater perception of wellbeing [8], and improved self-esteem [9].

### 1.1. Attention Restoration

It has been proposed that a combination of the human senses, modes of attention and cognitions during exercise may mediate the effects of green exercise [10,11,12]. One of the core theories behind the effects of green exercise is the Attention Restoration Theory (ART) [13]. The ART is based upon two types of attention (involuntary and directed). Directed attention requires focus and concentration involving cognitive control processes [10] (p. 1207). If overused, it can result in mental fatigue [14]. Indirect or involuntary attention is defined by Kaplan and Berman 2010 as “attention which requires no effort such as when something exciting or interesting occurs.” [15] (p. 46). The natural environment can promote the use of involuntary attention, which may provide recovery from mental fatigue [3]. Berman et al. (2008) [10] found that following a 50-min walk in nature, participants performed significantly better at the digit span backwards test, but this was not found following exercise in an urban environment. These results have been supported by Berto (2005) [16] and Hartig et al. (2003) [17]. Whilst this evidence highlights the capacity of exercise in natural areas to improve directed-attention performance, what has not been investigated is whether different forms of green exercise may alter these benefits when the intensity and duration are similar [18,19].

As highlighted by the ART, exercise in the presence of nature can provide a distractive stimulus for the human mind, requiring low levels of directed attention [13]. Researchers have theorised that exercise in a natural environment encourages a more externally focused, dissociative attentional style. This has been hypothesised to distract exercisers from feelings of over-exertion, thus resulting in increased levels of positive affect and mood [6,7]. Whilst an outdoor activity such as golf is conducted in a highly natural area, at a low intensity, it requires an associative attentional style when performing an action, planning tactics and assessing distances [20,21]. Consequently, it may be that the individual is not sufficiently distracted by the natural environment when conducting such an activity. The current study investigates differences between attention styles. Associative attention styles are defined in the literature by Masters and Ogles (1998) [22] as focus on the body, physical sensations and pain. Whereas dissociative attention was defined as focus on anything other than the body and internal sensations. As this definition was based around runners, the current study included focus on the task as an associative style of thought.

### 1.2. Golf and Wellbeing

Previous research examining thoughts during golf, demonstrated that participants often thought about the environment, but these were viewed as stressors, citing ‘wind’ and ‘trees’ [23]. Despite this, several studies have shown positive effects on wellbeing and mental health following golf [24,25]. It has been established that thoughts may differ based on the environment which an individual is exercising in [26]. However, whether cognitions differ between different forms of green exercise activities and how these alter the benefits obtained from green exercise requires investigation. Whilst green exercise studies have typically examined these effects based on walking, golf is comparable in intensity [27].

The psychological benefits that occur from playing golf can be attributed to a number of factors. The social element allows participants to meet and socialise with friends whilst playing sport [28]. Golfers conduct low-intensity exercise, covering distances upwards of 10 km in a round which can promote increases in mood [24]. However, many golf studies have not considered whether the natural environment further enhances these benefits. There is also a lack of green exercise studies examining golf despite it being an apparent avenue of investigation, due to the natural environment the activity is conducted in.

### 1.3. Rationale

Previous green exercise studies have suggested the need for research in this area as it remains unclear what factors may be involved in determining the benefits of green exercise. Numerous studies have investigated the psychological differences between intensities, environments and time spent outdoors, although few have considered the effects which may occur from varying forms of green exercise. Rogerson et al. (2016) suggested “the need for identifying remaining gaps in green exercise where the focus on the activity is greater for example during games or sports” [18] (p. 12). This point was echoed by Han and Wang (2018) who stated, “green exercise studies need to investigate a range of physical activities, participants and environments.” [29] (p.12). In terms of the study design, researchers Bamberg, Hitchings and Latham (2018) [30] also highlight that future green exercise studies should look to use more qualitative research methods to increase understanding between the exercise and environment response.

### 1.4. Aims

The following study aimed to assess whether differences exist between conducting walking and golf in a natural environment. A secondary aim was to investigate whether benefits occurred within the respective groups following 30 min of green exercise.

### 1.5. Hypothesis

1. Based on the Attention Restoration Theory, the walking condition will exhibit more dissociative styles of attention and the golf condition, greater associative.
2. The walking condition will perform better at a directed attention test.
3. The golf participant’s performance on a directed attention test will be reduced as a result of using directed attention.
4. The walking condition will experience greater increases in categorical and dimensional affect.
5. Participants in the walking condition will perceive the activity to be easier due to using greater levels of dissociative thoughts.

## 2. Materials and Methods

### 2.1. Research Design

The current study used a mixed method, between-groups design to compare the responses to green exercise between walking and golf. Tashakkori and Teddlie (2010) [31] state, through adopting a mixed-method study design, this may strengthen the research and reduce the likelihood of missing important information. Each activity was conducted for 30 min. This time duration was selected to meet the current daily physical activity guidelines [32,33]. This duration has also been shown to be sufficient to allow positive changes in mood [34,35,36,37]. Participants were required to walk at a self-selected intensity, increasing the ecological validity of the study through replicating a real-world experience. Walks were completed at a rural local park and a golf course. Golf participants were instructed prior to data collection to use a trolley to transport their golf clubs to reduce supplementary fatigue. Data collection took place from late October until mid-May. During the data collection period, the weather was typically cloudy, and temperatures ranged from 0–19 °C. No sessions were cancelled due to inclement weather. Trials were completed at the same time of day (+2 h).

### 2.2. Study Sample

A total of 20 participants were recruited to take part in the study, equally divided between the two modes of green exercise (golf condition: 10, walking condition: 10, mean age = 29.6 years SD = 7.14, Male = 17 Female = 3). Participants in the walking condition had an average body mass index (BMI) of 22.46 and were deemed active based on the Leisure time exercise questionnaire (LTEQ) responses. The majority of walking participants only conducted recreational leisure exercise. Golf condition participants were members of a golf club in Scotland and had a range of handicaps (3–28), experience in the sport ranged from 1 year to 15 years and time spent training each week was varied (4–28 h per week). Participants in the golf condition had a BMI of 24.91 and were active based on LTEQ responses. All participants were British white, middle class, had similar education levels and were free from any contraindications to exercise. The study sample was estimated through using a priori power calculation using the software G-power version 3.1.9.4 (Heinrich-Heine-Universität Düsseldorf, Düsseldorf, Germany) [38]. The calculation used an alpha value of 0.05, with a moderate effect size 0.5—as used in previous studies [6]—and a power value of 0.8. The results of the calculation highlighted a study size of 12. This sample size was comparable to similarly conducted studies [6,11,18,39]. Due to the performance experience required to effectively participate in golf, randomisation was not possible and, therefore, participants were self-selected into the two conditions.

### 2.3. Measures

The researcher collected data using both quantitative and qualitative research methods to allow a robust investigation into the differences across the two forms of green exercise. Self-reported measures were used to assess (a) affect and (b) ratings of perceived exertion. Objective data was collected in the form of step counting via a pedometer to allow the intensity of the two conditions to be compared, attention styles and thoughts during the specific activity. Directed-attention performance was collected via a short working memory test.

#### 2.3.1. Baseline Measures

Baseline physical activity levels were measured using the Pre-Activity Readiness Questionnaire (PAR-Q) [40] and the LTEQ [41].

#### 2.3.2. Affect

Affect is defined as an individual experience of any valenced state which can be altered by “bodily sensations, cognitive appraisals, and/or instrumental responses” [42] (p. 44).

The Exercise-Induced Feeling Inventory (EFI) [43] is a 12-item multidimensional scale measuring the degree to which participants experience four select affective responses: revitalization, physical exhaustion, positive engagement, and tranquillity. A number of previous green exercise studies have used the questionnaire to measure mood after acute bouts of exercise [6,44,45]. The EFI was selected due to its ability to detect changes in mood states following acute bouts of exercise [46]. Further, it has been noted that the EFI accurately assesses alterations in mood following changes in the exercise environment [43].

#### 2.3.3. Feeling Scale [47]

The Feeling Scale (FS) is a single-item, 11-point scale that assesses an individual’s feelings of pleasure–displeasure. Participants rate how they feel at the present time on a scale ranging from −5 = very bad to +5 = very good. Previous studies have found that the FS provides adequate levels of convergent and discriminant validity [48].

#### 2.3.4. Felt Arousal Scale [49]

The Felt Arousal Scale (FAS) is a 6-point scale measuring the activation dimension of basic affect. Participants rated their current level of perceived activation using a scale ranging from 1 = low arousal to 6 = high arousal. Previous green exercise studies have used the FS and FAS to examine changes of affect during bouts of physical activity [6,39,50].

#### 2.3.5. Directed Attention

The digit-span backwards test [51] assesses directed-attention abilities as participants must move items in and out of their attentional focus [52]. This is a major component of short-term memory [53]. The test was completed by participants before and after each session. The Digit Span Backwards Test has been used in various green exercise studies that have examined the restorative effects of nature in assessing participants short-term attention [10,12,54].

#### 2.3.6. Rating of Perceived Exertion (RPE) [55]

The participant’s perceived exertion was assessed using Borg’s 15-point RPE scale [55], which ranges from 6 to 20. A verbal response ranging from 6 = no exertion at all to 20 = maximal exertion indicates the participants current perceived exertion.

#### 2.3.7. Cognitions and Thoughts

The terms cognition and thought are used interchangeably throughout this study. Concurrent verbal reports were used for recording thoughts during the two green exercise activities. This technique involves participants verbalising thoughts that passed through their minds into a recording device [56]. Thoughts were collected when instructed by the researcher during three points of the study (10, 20, 30 min), as used in previous studies [42].

### 2.4. Procedure

Following gaining ethical approval from the University ethics board (ETH905), participants were recruited through posters placed on notice boards around local golf courses and community areas. On the day of testing, the participant first completed the PAR-Q and L-TEQ to ensure readiness to exercise. The EFI, FS and FAS questionnaire were conducted to assess affect. The digit span backwards test was then administered by the researcher to assess attentional focus. Finally, the participant was then fitted with the recording device and pedometer. The researcher followed behind the participant to avoid any unnecessary distraction. At three intervals (10, 20, 30 min), the researcher requested the participant to speak into the recording device for ten seconds about their current thoughts. At this point, the researcher also collected the level of perceived exertion. Midway (15 min), the FAS and FS were collected. To ease the data collection, the researcher held sheets detailing the RPE, FS, and FAS levels and participants verbalized their responses and/or pointed to the appropriate value. Following the 30 min elapsing, each group stopped immediately and completed the EFI, FS, FAS, RPE and the digit span backwards test.

### 2.5. Data Analysis

Quantitative analysis was conducted via IBM SPSS Version 23 software (SPSS Inc., Chicago, IL, USA). Quantitative analysis compared between-group and within-group differences. The non-parametric, Mann–Whitney U test was used to assess whether there was a significant difference in the intensity (steps), and frequency of associative and dissociative thoughts experienced during the two activities. Differences between groups on the digit span backwards test were analysed via gain scores (post-test–pre-test) in an ANOVA with outdoor condition (walking vs. golf) as the independent variable. Between-group differences in the EFI were assessed via split plot ANOVA 2 (golf/walk) × (time: pre/post). For RPE and the valence and activation dimensions of affect, a split plot ANOVA was used to assess differences during exercise, × 2 (golf/walk), × 3 (Time: 10, 20, 30 min). Bonferroni post-hoc tests were conducted between the conditions and times. Data was collapsed across the two conditions, allowing Mann–Whitney U tests to be conducted to examine where statistical significance existed and at what time points between the conditions.

#### 2.5.1. Within-Group Differences

The Wilcoxon signed ranked test was used to compare mean differences (pre–post) within-groups for categorical affect measures and directed attention performance to assess the change following each condition. A repeated-measures ANOVA was used to test for differences between rating of perceived exertion (RPE) and the valence and activation dimensions of affect (FS, FAS) × 3 (Time: 10, 20, 30 min) within groups. Kendall’s Tau correlational test was used to investigate whether the use of dissociative cognitions influenced performance of the digit span backwards test and perceived exertion. Finally, Kendall’s Tau was conducted on the intensity (steps) and RPE.

#### 2.5.2. Cognitions

Collected data was transcribed verbatim and each individual transcript was subject to checks for relevance and consistency. Transcribed data was analysed through coding the information into thoughts. Individual “thoughts” were termed a unit of analysis and coded as associative or dissociative-internal thought as described by Morgan and Pollack (1977) [57]. An associative thought was said to involve attention focusing on stimuli related to the outdoor condition. Such examples included bodily sensations and/or factors related to the task being performed. A dissociative thought was said to involve attention focus of non-task related stimuli such as daydreaming or scenery [26].

#### 2.5.3. Qualitative Analysis

From the transcribed data, a thematic analysis was conducted. Thematic analysis is defined by Braun and Clarke 2006 [58] (p. 6) as a qualitative research method for “identifying, analysing and reporting themes” within a study’s collected data. Braun and Clarke (2014) [59] also highlight this as a valid and reliable analysis method for applied health research. The analysis of the collected data was driven towards comparing the responses from the two groups; therefore, the researcher used an analyst-driven, deductive analysis [60] at the semantic level. Validity of the thematic analysis was confirmed through independent assessment from two researchers (S.M., S.A.M.). Thematic analysis consists of six phases as outlined by Braun and Clarke (2006) [58]. The six steps of the conducted thematic analysis are detailed below.

1. Familiarisation with the data.
2. Generating initial codes.
3. Searching for themes.
4. Reviewing themes.
5. Defining and naming themes.
6. Producing the report.

## 3. Results

Table 1 (below) outlines the characteristics of the sample used in the current study.

### 3.1. Intensity and Perceived Exertion (RPE)

**Figure 1:**
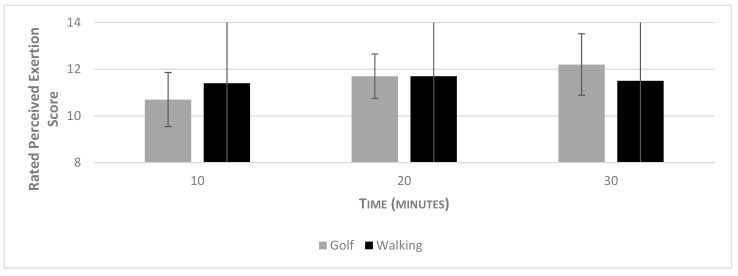
Rating of perceived exertion scores by time and outdoor condition.

Results of the Mann–Whitney U test revealed intensity significantly differed between the two groups, with the walking group conducting more steps (*p* < 0.01); effect size = 0.64 (moderate). However, a Mann–Whitney U test revealed non-significant differences between the two groups for RPE (*p* = 0.337). When comparing perceived exertion between groups, the Mann–Whitney U test revealed no significant differences between the two conditions at time point 10 vs. 10 min (*p* = 0.67), 20 vs. 20 min (*p* = 0.74), 30 vs. 30 min (*p* = 0.08). In terms of within-group differences, a repeated-measures ANOVA revealed a significant difference of perceived exertion over time within the 30 min in the golf condition (F2,18) = 15.00, *p* < 0.01), effect size = 0.625). Post-hoc, pairwise comparisons adjusted for Bonferroni revealed significant differences in perceived exertion between times; 10 vs. 20 min (*p* = 0.025), 10 vs. 30 min (*p* = 0.01). For the walking condition, no significant difference in RPE over the 30 min was found (F1,204,10.84) = 0.455, *p* = 0.550. No significant correlation was found between steps and RPE. A graphical representation of the perceived exertion and intensity values for both groups are displayed below in Figure 1.

### 3.2. Cognitions During Activity

Walking participants exhibited significantly more dissociative cognitions than the golf participants (*p* = 0.02). However, with regards to correlation, only the golf group showed a statistically significant positive correlation which was between dissociative thoughts and RPE (τb = 0.659, *p* = 0.01). Table 2 (below) demonstrates the amount of recorded cognitions during each of the green exercise activities. 

### 3.3. Directed Attention Performance

A Wilcoxon signed rank test showed that performance on the digit span backwards test following the 30 min activity significantly improved for the walking group (Z = −2.40 *p* = 0.01). For the golf condition, performance was non-significantly reduced in performance (Z = 0.00 *p* = 1.00). No significant difference between the pre-exercise directed attention scores was found (*p* = 0.26). The results are presented below in Table 3.

### 3.4. Affective Responses

Table 4 (above) shows affective responses from the golf and walking participants. A repeated-measures ANOVA demonstrated Feeling Scale (FS) responses for the golf condition increased but did not reach significance over the thirty minutes (F (2,18) = 1.51, *p* = 0.246). In terms of the walking condition, the repeated-measures ANOVA found that affective valence differed significantly across the 30 min session (F (1.26,11.40) = 7.68, *p* = 0.01). Bonferroni post-hoc analysis revealed significant differences in responses between Pre vs Post (*p* = 0.04). When comparing differences between the two groups, a split plot ANOVA 2 (golf/walk) × 3 (time; pre, 15 min, 30 min) was used to identify whether differences existed. There was no significant difference for affective valence between the two groups (F (1.41, 25.43) = 2.56, *p* = 0.12 d = 0.39). Results revealed significant differences for time (F (1.41,25.43) = 6.27, *p* = 0.01 d = 0.76). No significant differences between or within groups were seen for the analysis of the Felt Arousal Scale.

### 3.5. Categorical Affect

#### 3.5.1. Revitalisation

In the golf condition, it was found that there was no significant difference between the pre and post scores (*p* = 0.79). For the walking condition, it was found that there was a significant increase in revitalisation pre/post (*p* = 0.005). Split-plot analysis of variance between the groups revealed significant differences via a 2 × 2 ANOVA (F (1,18), 39.6, *p* = 0.00).

#### 3.5.2. Positive Engagement

In the golf condition, it was found that there was no significant difference between the pre and post scores (*p* = 0.75). For the walking condition, it was found that there was a significant increase in positive engagement scores pre/post (*p* = 0.05). Split-plot analysis of variance between the groups revealed significant differences via a 2 × 2 ANOVA (F (1,18), 5.08, *p* = 0.03).

#### 3.5.3. Tranquillity

In the golf condition, it was found that there was no significant difference between the pre and post scores (*p* = 0.14). For the walking condition, it was found that there was a significant increase in tranquillity scores pre/post (*p* = 0.01). Split-plot analysis of variance between the groups revealed no significant difference via a 2 × 2 ANOVA (F (1,18), 1.03, *p* = 0.32).

#### 3.5.4. Physical Exhaustion

In the golf condition, it was found that there was a non-significant increase between the pre and post scores (*p* = 0.85). For the walking condition, it was found that there was a significant decrease in physical exhaustion scores pre/post (*p* = 0.02). Split-plot analysis of variance between the groups revealed significant difference via a 2 × 2 ANOVA (F (1,18), 6.80, *p* = 0.01).

### 3.6. Qualitative Analysis

**Figure 2:**
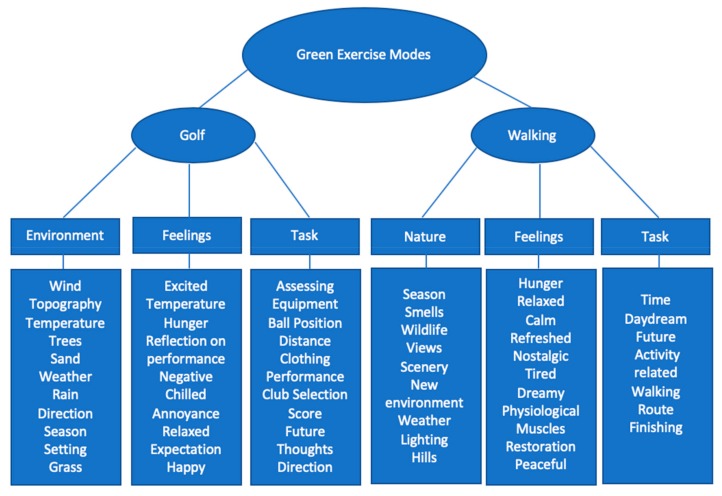
Codes and themes from the thematic analysis.

Results from the thematic analysis demonstrated a number of interesting similarities and differences between the two forms of green exercise. To demonstrate the responses in which the themes were developed from, the evidence and reasoning for the development of each theme is described below. Figure 2 (below) displays the themes, sub-themes and codes in a hierarchy framework.

As a deductive analysis was conducted on the collected data, the main themes were generated primarily via the predetermined research question. The main themes were labelled as the two green exercise activities (golf and walking). The two main themes consisted of several similar sub-themes; golf (task, environment, feelings) and walking (task, feelings, nature). The primary aim of the thematic analysis was to determine whether differences existed in participants experience and perception of the two green exercise activities. Further, to provide causality and understanding to the quantitative findings.

#### 3.6.1. Golf

Within the main theme of golf, there were several interesting findings from the qualitative data collection. Each sub-theme interlinked, and it was evident that the task or outcome of an action directly influenced the participants’ perception of the environment. Further, the task consequently altered participants’ feelings during the activity based on their perceived success or failure of the varying tasks associated with successful performance in golf. It may also be considered that expectancy effects play some role in mediating the benefits of green exercise, for example, if a golfer expects to perform well and does not meet the desired level, it is likely that negative feelings such as stress and frustration may negate any potential benefits from green exercise. Moreover, the participants skill level may have influenced what degree of thoughts were related to performance. Research has shown highly skilled performers will analyse more variables and stimuli than lower skilled ones [21].

##### Sub-Theme: Environment

Participants in the golf condition consistently acknowledged elements of the environment (sand, trees, wind, grass, colours). However, these were related to influencing performance rather than aesthetics. This was expected as participants in this group are consistently interacting and assessing natural features during play. The colour green was frequently cited; however, this was typically in relation to the putting surface in golf and not the sights of the natural environment. It might be considered that these participants, rather than being distracted by natural stimuli which can promote restoration, in fact perceived natural elements instead as obstacles which caused negative psychological responses. Some examples of this are demonstrated below. Finally, what motivates an individual to seek out the various forms of green exercise may also potentially alter what degree of benefits are attained. If the participant conducts a form of green exercise to get outdoors and enjoy the activity, it is likely that they will take in far more stimuli from natural sources, opposed to the participant who is driven by achieving a specific performance goal.

Participant 2: “*I’m thinking about the wind direction coming right into my face and the fact that if I hit a wood it’s going to rise up high, so instead I am going to play a low 3 or 4 iron which will leave myself with a full shot into the green.*”

Participant 10: “*I’m thinking about my last shot which I just put into the trees and then the sand, which annoyed me but overall it was a good half an hour play. I just need to start playing more often to get better.*”

Participant 9: “*Just thinking about my approach shot onto the green and how I’m going to judge the distance of the putt and starting to read the line whether it’s going to go left to right which I think it is by walking up to the green now, also the weather has got a lot better which is also making me feel better.*”

##### Sub-Theme: Feelings

The participants in the golf condition outlined a range of feelings during the activity. Contrastingly, to what green exercise studies have previously shown, participants stated negative emotions such as annoyance and stress. Whilst these responses may support findings from the quantitative results that mood and affect were not significantly improved, anecdotal responses from the golfers still highlighted several positive responses such as relaxation, excitement and happiness that occurred during and after play, which is demonstrated in the responses below. These highlight the wide spectrum of emotions during task-related forms of green exercise. It appears that the outcome of a task will alter the psychological response depending on the perceived outcome.

Participant 10: “*Right, I am thinking about it’s obviously quite hot, I am pretty tired after climbing up the hill at the second, so I am just thinking about slowing down and catching my breath back before I get to my ball which I can see already. So I am already kind of looking at the next shot. I’ve got an idea in my head of what I am going to do and what club I am going to use.*”

Participant 3: “*I’m walking along thinking about my next shot, and it’s a really nice day, it’s sunny and I’m feeling relaxed, just going to chill out after golf.*”

Participant 6: “*Just holed a massive putt, I feel really relaxed and was excited with the putt, em I’m getting onto the next hole and going to make it bigger and better.*”

##### Sub-Theme: Task

The majority of responses recorded discussed some element of performance of the task. Participants frequently voiced thoughts regarding the location of their ball, the distance from their ball to the identified target and which golf club they would select to perform the desired action. Consequently, as noted previously, the task which the participants were conducting altered their perceptions of the environment; for example, trees were viewed as an object to stop an individual achieving their goal. Further, as a result of poor execution of a task, participants would describe negative feelings. On the contrary, if a participant performed well at the given task, positive emotions would be displayed. The responses below demonstrate how the task and outcome may have influenced thoughts. With this performer, their attention will be largely on the task, further previous research has shown a narrow focus of attention is required for peak performance in golf [61] and thus, external stimuli such as nature may be ignored. The following responses demonstrate how participants were consistently thinking about the activity.

Participant 9: “*I am thinking about my putt just about to come up, I’ve got a good birdie chance, I’ve just parred the first couple holes so it would be good to get the first birdie of the round, just thinking about that really.*”

Participant 8: “*Just basically thinking what club to use to approach this next green, where the flag is positioned, em it’s at the far left and I’m at the right of the fairway so it gives me a good approach shot into it and also there is clear skies now without the wind and rain.*”

Participant 1: “*Ok again just thinking about the shot I am about to play, emm I hit the fairway off the tee and now about to approach from about 50, 60 yards. So just looking to hit the ball low and roll it onto the green.*”

#### 3.6.2. Walk

Participants responses in the walking group were similar to those reported in previous green exercise studies [19,39,62]. Participants responded with thoughts related to positive feelings or emotions as a result of conducting a walk in a natural environment. The sub-themes overlapped in some instances, for example, participants would note that elements of nature resulted in positive feelings or thoughts related to the task. However, there were negative elements of nature (wind, temperature) and physiological responses such as sore legs or muscles, which have been under-examined in previous research, which participants highlighted as evoking negative responses and highlights the importance of using qualitative research methods in green exercise studies.

##### Sub-theme: Nature

As hypothesised, participants in the walking group tended to be increasingly focused upon the environmental settings in which the activity was taking place. The sub-theme was comprised of several different codes with participants frequently noting affordances offered from the natural environment such as weather conditions, wildlife, temperatures and surfaces. Participants also noted lighting, season and smells they experienced during the walk.

Participant 3: “*I’m thinking about the surroundings of the park, I’m aware of the birds and em, yeah just out walking.*”

Participant 5: “*I’m thinking about how nice a day it Is, how green and pretty the fields are and yeah I’m feeling peaceful.*”

Participant 9: “*I guess feeling a bit warm, em that’s kind of really all I am fixated on. And yeah just looking around, thinking about how new this place looks and feels, when I was growing up around here it was generally fields which is quite an amusing thought but yeah that’s about it.*”

##### Sub-theme: Feelings

Participants noted positive feelings such as relaxation, peacefulness, increases in affective valence, mental restoration and even nostalgia during walking. However, some participants, whilst walking, also noted feelings of fatigue, reduced body temperature and sore muscles. The results demonstrate that whilst walking in nature can promote increases in mental parameters, individuals can still experience physiological discomfort, which is rarely highlighted in green exercise studies. Further, participants stated they felt they had a “clearer head”, which the author interpreted as a form of mental restoration. The Attention Restoration Theory [13] notes that as a result of using indirect attention such as viewing nature, this provides such benefits and thus the study provides support for this.

Participant 2: “*Just thinking it’s a lovely walk, feeling quite energised, there are lovely surroundings, em if I didn’t have work I could do this every day and anytime especially in the winter.*”

Participant 2: “*Ok so I’m feeling good, I actually feel like I want to keep going. I could do another tour of the hill, I liked the uphill bits and then the downhills as it gets the heart going but it’s just made me feel good, whilst thinking about a lot of things such as work and the nice smells.*”

Participant 4: “*Em, yeah legs are feeling a little sore, but its been a nice bit of exercise, it’s cleared the nose as I have a cold, em that’s it, been a lovely day for a walk.*”

##### Sub-theme: Task

Contradictory to what was hypothesised, participants conducting the walking still noted several thoughts directly related to the task. However, it was possibly the least reported of the six sub-themes. It was hypothesised that as a result of walking in a natural area, participants would exhibit significantly less associative thoughts (to do with the activity) and a greater focus would be placed on the surroundings and thoughts away from the activity. Participants thoughts regarding the walk were related to exercising and physical activity, the duration of the walk and on occasions, contentment to be finished the walk. Walking group participants would also discuss the route and also how the walk allowed them to daydream and plan ahead for the rest of the day.

Participant 10: “*Yeah it’s been a really enjoyable walk actually, I feel like I’ve had a good bit of exercise and weather has been brilliant, so yeah it’s been enjoyable so far and eh looking forward to going back now.*”

Participant 3: “*Yeah so I’m just thinking about how I’ve enjoyed getting out of the house for a bit, it’s been a fine half an hour walk, I’ve seen some nice sights and happy with it to be over and get home now.*”

## 4. Discussion

The aim of the present study was to assess whether differences existed between two forms of green exercise. A secondary aim was to examine whether within-group differences were evident for the golf and walking groups, respectively. Based on previous evidence, it was hypothesised that the group conducting the walking would use greater levels of dissociative cognitions and thus exhibit lower levels of RPE, greater increases in positive affect and improve directed attention performance. In agreement with this hypothesis, participants in the golf condition exhibited significantly less dissociative thoughts than those in the walking condition. Predictably, it was also found that the golfing group used more associative thoughts, but this did not reach significance. Consequently, participants in the walking group demonstrated significant improvement in a working memory task following thirty minutes of green exercise. The golf condition as expected demonstrated a reduction in performance. It was possible that the use of directed attention, reduced performance in the digit span backwards test in the golf condition and this was supported through recording and measuring cognitions.

Previous studies have investigated directed attention performance within natural environments [10,14,16,18,63]. Berman et al. (2008) [10], found that following comparing walking in a natural vs urban environment, participants scores improved greater in the natural setting compared to the urban. The results were in agreement with findings from the walking condition but not the golf condition. This highlights that despite golf being conducted in a highly natural area, the use of directed attention during the activity may reduce potential benefits. There are several reasons why directed attention performance may have improved in the walking condition besides from the difference in activities. As the majority of golf participants were members of the course where the study was undertaken, it may have been that the environment was not sufficiently fascinating enough to distract the participants that exercised in it regularly. Kaplan and Kaplan (1989) [64] highlight the natural area must provide sufficient ‘fascination’ in order to promote increases in indirect attention. These findings may provide further support for the ART, demonstrating that when directed attention is used in a natural environment, restoration is not evident.

Participants in the walking group demonstrated a significant decrease in the physical exhaustion subscale, whereas the golf condition in fact showed a non-significant increase. Researcher’s Han and Wang (2018) [29] raise an interesting point regarding attentional focus. They state if an individual exercising in nature shifts attentional focus away from the environment, the physical and psychological benefits may be removed. Further, they note benefits from nature can be reduced due to long duration, high-intensity or high workloads due to focusing on internal attention. Support for this finding has been shown in previous studies, LaCaille et al. (2004) [65] found that runners that used greater dissociative styles of thinking during exercise, reduced their feelings of perceived exertion. Previous studies have shown playing golf requires high levels of directed attention and concentration [20,66]. In terms of the exercise environment, previous studies have found that exercising in natural areas may promote a more external focused attention style, which may cause decreases in RPE in comparison to indoor exercise even when participants self-selected higher exercise intensities outdoors [65,67]. In the current study, no significant findings were shown for RPE within either condition; therefore, no firm conclusions can be drawn towards attentional focus and level of perceived exertion.

With regards to mood and affect, it was found that following the 30 min, there were improvements in different components of affect within both conditions. When examining the categorical affective responses from the EFI, participants in the walking condition reported significant improvements in all four subscales. The golf condition showed no significant improvements for any of the four subscales. It can, therefore, be inferred that psychological benefits are altered in different forms of green exercise and benefits maybe greater from activities that require less concentration or focus. These findings contrast previous research that exercise in natural areas promotes improvements in affective states. A previous study by Blanchard et al. (2004) [26] investigated the role of cognitions in influencing affective responses found that only participants who exhibited high levels of dissociative cognitions experienced increases in revitalisation pre/post and decreases in physical exhaustion. These findings were in agreement with the current study, which found the golf condition who predominately used associative thoughts, experienced decreases in revitalisation and increases in physical exhaustion. These findings support that the type of thoughts that participants experience during the respective activities may influence feelings and mood post-exercise. With golf being a performance-based activity, it may also be that feelings were correlated with how the player felt they were performing; however, further research is needed on different activities to confirm this.

Finally, it was hypothesised that as a result of conducting green exercise, both conditions would experience increases in pleasure–displeasure and activation. As hypothesised, the results demonstrated a significant, positive pre-test to post-test shift for the walking group and non-significant increase for the golf condition. There were no significant differences between groups. However, with regards to the perceived activation measure, both conditions non-significantly decreased over the 30 min. The results of the current study are in contrast to the majority of previous studies which have used the FAS to examine the role of physical activity and the natural environment in influencing affective states [6,39,68]. Referring to the study by Focht (2009) [6], it was found that when examining differences between outdoor and laboratory walking, responses from the FS and FAS increased in both conditions. In the current study, both conditions demonstrated decreases in perceived activation. Despite contrasting previous research, these results may suggest that following the 30 min activities, participants felt more relaxed or calm.

### Qualitative Analysis

The thematic analysis demonstrated distinct differences in thoughts and feelings experienced during the respective green exercise activities. Despite both groups exercising in highly natural areas, there was a clear dichotomy between how each group experienced and perceived the different activities. Each group had similar sub-themes generated from the collected data; however, the codes in each sub-theme were contrasting.

During the recordings, participants in the walking condition tended to discuss stimuli such as the weather, wildlife, the scenery and lighting, which were noted as interesting features during the walk. These affordances provided by the natural environment produced several positive psychological responses to physical activity. Feelings such as restoration, nostalgia, peacefulness, increased energy and relaxation were all noted as positive outcomes from walking. As hypothesised, walking participants rarely acknowledged thoughts relating to the task (associative cognitions). Much of these elements directly related to the Ecological Dynamics Theory [69]. The theory states affordances and stimuli offered by nature may promote psychological improvements. Results of the current study provided similar responses to those stated in the theory, providing support for it.

Contrastingly, when asked to verbalise their thoughts, participants in the golf condition frequently displayed thoughts relating to the task (associative cognitions). Participants cited aspects such as score, shot selection, distance and direction assessments and factors that they would like to improve related to the task. These qualitative findings provide further evidence as to why performance on the digit span backwards may have reduced and why no affective improvements were seen within golf participants due to continual use of directed attention. Therefore, with regards to the Attention Restoration Theory [13], participants were continually using associative cognition styles and thus, indirect attention was not given sufficient time to recover, consequently benefits of green exercise may have been reduced. It may be possible that golf participants’ focus was heavily on performance. With reference again to the Ecological Dynamics Theory [69], in terms of the golf participants when considering the sub-theme ‘environment’, the golf participants noted elements of nature as constraints to successful performance, citing wind, trees, length of grass and sand as barriers to performing well. As a result, it may be that participants in the golf condition negated any potential benefits offered from natural features due to their altered perceptions of such elements.

When performing such an activity as golf, the focus is clearly centred around performance of various techniques [66] and resulting negative feelings such as stress, anger and tiredness can occur when the outcomes do not meet intended expectations. The results of the current study were in agreement with previous studies which examined the thoughts by golfers [33] who found environmental elements to be highlighted when participants viewed the environment as a negative aspect to performance. Whilst this was somewhat evident in the current study, contradictory to this some participants still acknowledged positives from the environment the activity was performed in. Participants in the current study noted feelings such as relaxation and feeling ‘chilled’, happiness and excitement. However, overall it would be fair to state that golfers perceived the environment as a negative. Contrastingly, walkers acknowledged environmental stimuli such as wildlife, sights and weather, as positives which made them feel calm, relaxed and happy. Participants in the golf group cited various positive and negative psychological and physiological feelings during the 30-min. Moreover, participants in the golf condition highlighted physiological feelings such as tiredness, these anecdotal responses provide further support for the ART in that when conducting activities such as walking in which the participant conducted significantly greater levels of dissociative cognitions these perhaps distracted the walkers from feelings of exertion as highlighted in a study by Focht (2009) [6].

Responses from participants in the walking group were typically as seen in previous green exercise studies [6,19,62]. However, due to the lack of qualitative research conducted in this research field, comparisons were not possible. Further, there has been a lack of green exercise studies examining different forms of activities and, therefore, comparisons were not possible. The findings, however, support quantitative findings from previous studies which have shown that during 30 min walks, participants’ mood and mental restoration was improved [34,36,37]. From the results of the study, it is clear to see that when given a performance-based task in a natural environment, focus can override the potential benefits offered from green exercise. Further, even when in a highly natural area, poor performance can alter the mood or feelings of the individual.

In terms of promoting and prescribing green exercise to the public to prevent and manage mental health conditions, it may be fair to acknowledge that specific types of green exercise are not as consistent for providing benefits due to focus being placed on performance outcomes rather than enjoyment and taking in nature. However, further research is required to confirm this in different types of sports and activities conducted in natural areas. Outside of the green exercise research paradigm, there has been a lot of research into coping strategies in golf. Based on the evidence it may be that researchers have overlooked the simplest form and that would be to alter the participants perceptions of the natural features on the golf course. This could be an interesting avenue of research due to green exercise research demonstrating reductions in stress and anxiety when viewing natural scenes.

## 5. Future Research

Due to the range of green exercise activities that are conducted in a natural area, future studies should investigate how different cognitions, affect and directed attention responses differ across these. Whilst not a weakness of the current methodology but more of a generalisation of the green exercise literature, using a more chronic timescale would allow the researcher to understand whether the effects seen are consistent over time or simply acute. In terms of the demographics of the employed sample, only male golfers volunteered. Unfortunately, this may be representative of the current demographics of golf courses around Britain [70], but future studies should seek to include male and female participants. Another interesting investigation might be to compare green exercise activities which require varying levels of focus in comparison to indoor environments to address if potential supplementary benefits still exist.

## 6. Conclusions

To the authors knowledge, this was one of the first studies to investigate how the psychological benefits of green exercise differ across activities. The findings from the current study add support for the Attention Restoration Theory, in that the type of thoughts participants exhibited may have contributed to alterations in affective responses and directed attention performance. Whilst there are clear mental and physical health benefits to conducting golf in a natural environment, overall, it appears as if the benefits from green exercise are somewhat reduced when conducting different modalities which require different levels of attention and performance outcomes. Further, as a result of conducting golf in a highly natural area, participants’ perceptions of the environment are altered and environmental features are viewed as a hindrance to performance and not aesthetically pleasing, promoting indirect attention; however, further research is needed to confirm this.

## Figures and Tables

**Table 1:** Descriptive Statistics of the selected sample and exercise responses.

Measure	Mean	SD
Age (years) Whole sample	29.6	7.14
Walking Group (age years)	34.7	15.5
Golf Group (age years)	24.6	2.88
LTEQ (session/week)		
Strenuous	Golf: 2.9	1.73
	Walking: 1.7	1.34
Moderate	Golf: 3.8	1.93
	Walking: 4.5	1.72
Mild	Golf: 4.6	2.27
	Walking: 6.6	0.84
Intensity (steps)		
Walking Group	3116.2	367.29
Golf Group	2242.7	306.41
RPE (mean total)		
Walking group	11.4	0.21
Golf group	11.5	0.76

Note: Mean; SD = Standard Deviation; LTEQ = Leisure time exercise questionnaire; RPE = Rate of perceived exertion.

**Table 2:** Frequency of associative and dissociative thoughts during activity.

Cognition Type	Golf	Walking
Associative	81	55
Dissociative	15	49 *

* significant difference between groups (*p* < 0.05).

**Table 3:** Digit Span Backwards Test performance.

Golf Condition Mean (SD)		Walking Condition Mean (SD)	
Pre	Post	Pre	Post
5.4 (1.51)	5.2 (1.87)	4.7 (1.95)	5.9 * (1.52)

* significant difference within groups (*p* < 0.01).

**Table 4:** Affective states for the two conditions.

Measure	Walking Condition		Golf Condition	
	Mean	SD	Mean	SD
Feeling Scale (FS)				
Pre	2.0	1.83	3.4	1.26
15 min	3.0	1.83	3.2	1.62
Post	3.5	1.58	3.9	0.99
Felt Arousal Scale (FAS)				
Pre	2.6	0.97	2.5	0.85
15 min	2.8	1.03	2.6	1.17
Post	2.4	1.51	2.2	0.92
EFI Subscales				
Revitalisation				
Pre	4.9	2.18	7.3	2.54
Post	9.5	2.01	7.1	2.73
Positive engagement				
Pre	7.3	1.77	8.1	3.03
Post	9.7	2.0	8.5	1.51
Tranquillity				
Pre	7.2	1.87	7.4	3.69
Post	9.5	2.55	8.6	1.9
Physical Exhaustion				
Pre	6.4	3.34	4.8	2.3
Post	3.1	2.42	4.9	1.91
